# Green digitization: Online botanical collections data answering real‐world questions

**DOI:** 10.1002/aps3.1028

**Published:** 2018-03-07

**Authors:** Pamela S. Soltis, Gil Nelson, Shelley A. James

**Affiliations:** ^1^ Florida Museum of Natural History University of Florida Gainesville Florida 32611 USA; ^2^ iDigBio Florida State University Tallahassee Florida 32306 USA; ^3^ National Herbarium of New South Wales Royal Botanic Gardens and Domain Trust Mrs Macquaries Road Sydney New South Wales 2000 Australia

Recent advances in digital technology, coupled with rapidly increasing interest in the creation and dissemination of digitized specimen data for use in broad‐scale research by botanists and other organismal scientists, have encouraged the development of a variety of new research opportunities in the botanical sciences (e.g., Page et al., 2015; Soltis, 2017). It is now increasingly possible to collect, use, re‐use, and share data more easily and effectively. With the advent of the U.S. National Science Foundation's Advancing Digitization of Biodiversity Collections initiative and the establishment of iDigBio (Integrated Digitized Biocollections; www.idigbio.org) as the national resource for specimen digitization and digital data mobilization, researchers now have access to ever larger and varied digital data sets for visualization, analysis, and modeling and have new opportunities for adopting “big data” strategies for facilitating discovery. The iDigBio portal alone now includes nearly 20 million botanical specimen records, a figure that is growing rapidly as new institutions share their data. In this special issue of *Applications in Plant Sciences*, which is based on symposium presentations at Botany 2017 (the annual meeting of the Botanical Society of America and affiliated societies) and the XIX International Botanical Congress, authors present a broad array of examples of the latest developments in botanical biodiversity research using digitized specimen data, including in the fields of genomics, conservation assessment, ecology, phenology, and taxonomic revisions. The papers present current trends in the proactive digitization of specimen data that occurs during the collecting and vouchering of specimens and field data; the tools, skills, and strategies needed for linking and visualizing botanical data; and innovative methods for digital discovery. This collection also highlights how digital data are being used in research that expands our understanding and conservation of plant diversity and the environment.

Although the source data for the papers in this collection are herbarium specimens, the topics extend well beyond systematics. Broadly integrative plant biologists will be interested in new approaches to using and re‐using specimen data—whether locality information for modeling or images for analysis of morphology and/or functional traits. More importantly, digitized herbarium data become even more valuable when linked to other data sources, such as environmental or genetic data. In fact, emerging cyberinfrastructure and new data sources provide unparalleled opportunities for mobilizing and integrating massive amounts of information from organismal biology, ecology, genetics, climatology, and other disciplines. Particularly powerful is the integration of phylogenies with specimen data, enabling analyses of phylogenetic diversity in a spatio‐temporal context, the evolution of niche space, and more. Such data‐driven synthetic analyses may generate unexpected patterns, yielding new hypotheses for further study. However, a major challenge is the heterogeneous nature of complex data, and new methods are needed to link these divergent data types. Ongoing efforts to link and analyze diverse data are yielding new perspectives on a range of ecological problems. Integration of plant phylogeny, distributions, traits, and ultimately genetics is permitting new perspectives on landscape‐level patterns of biodiversity, with implications for conservation and management of natural resources. Although many specific hypotheses may be addressed through integrated analyses of biodiversity and environmental data, perhaps the greatest value of such data‐enabled science will lie in the unanticipated patterns that emerge.

The papers in this collection capture some of the diversity of the emerging themes that can be addressed via use of digitized herbarium specimens. The authors address the broad range of research that can be facilitated by analysis of digitized herbarium specimens; limitations and bias of digitized specimens for certain avenues of research; future digitization and training needs; the role of globally unique identifiers (GUIDs) in integrative research involving herbarium specimens and other sources of data; digitization workflows that incorporate field, museum, and data mobilization components; the use of deep learning in specimen identification from images; the development of a standardized workflow for scoring plant phenology from herbarium specimens; the use of aggregated digitized data for fungi in generating a comprehensive mycological flora (or mycoflora) for North America; the role of digital images in education and public outreach; the effective contributions of citizen scientists of all ages to hypothesis‐driven research; and the need for effective, comprehensive, and accurate tracking of data use for understanding the impact of digitized collections.

Noting the centuries of exploration that have yielded the global span of the world's herbaria, James et al. (2018) provide an overview of how open, digitized, aggregated botanical data can be used to document global change, predict future impacts, and drive biological and environmental remediation. Herbarium data—from the information in labels to data that can be extracted from images—have an increasing role to play in analyses of temporal and spatial change in community composition and structure. Moreover, patterns identified via analyses of herbarium specimens can form the foundation for conservation, rehabilitation, and restoration efforts of not only single species but entire communities. However, collections data—whether plants, animals, or fungi—may not always be research‐ready. James et al. address the fitness for use of herbarium data in basic and applied research, noting that taxonomic, spatial, and temporal limitations may hamper the usefulness of herbarium data for specific questions. Fortunately, research efforts addressing issues of data quality, uncertainty, and bias are providing guidance for assessing limitations for specific uses and for ameliorating the effects. Given the enormous potential of herbarium data for research in systematics, ecology, conservation, and global change, the authors cite the need for greater global advocacy for collections, from curation of physical specimens to digitization to online publishing of digitized data. Future work to enhance digital herbarium collections through digitization of other resources, such as field notes, libraries, etc., and to develop tools for discovery, visualization, analysis, and communication is needed. Key to innovative and effective use of digitized herbarium data will be skills training for the next generation of botanical researchers.

The assignment of GUIDs to facilitate the tracking, linking, and discovery of biodiversity specimens across the internet has been a hotly debated subject. Although the majority of biodiversity informaticians agree that the use of unique identifiers is essential, controversy remains about which types of identifiers are best, the most appropriate Darwin Core field in which they should be published, strategies for resolving identifiers to physical specimens across the internet, and effective implementation strategies for the wide variation in biodiversity collections storage, management, and digitization. Nelson et al. (2018) narrow the scope of this debate to the implementation of GUID assignments to the digitization and mobilization of herbarium specimen data. They review the types of GUIDs in current use and strongly recommend that GUID values be associated with all specimens and included in all digital records of those specimens. They address the lack of a universal, community‐supported resolver for GUID values and offer guidelines and recommended practices for minting, managing, and sharing GUIDs for herbarium specimens.

Contreras (2018) brings a paleobotanical perspective to this special edition, highlighting the important role researchers can play in incorporating collection, digitization, analysis, curation, and data mobilization into an integrated research and digitization pipeline. Although she emphasizes that the workflow and pipeline presented may be especially useful in smaller institutions with limited staff or when images and other digital data are integral to the research project, the protocols she outlines may have broad applicability to researchers and other staff working in larger collections, as well as to those in non‐paleontological collections, including herbaria. Her workflow incorporates three components—field, museum, and data mobilization—that are often temporally and spatially separated in current practice. As a result, the paper brings a clear museum perspective to the research process, with the museum phase serving as a transition during which specimens are organized, data are bridged from field to museum, and the preparation of a museum workspace designed to facilitate these steps. Contreras' paper offers an important viewpoint on the ways in which research, collections management, digitization, and curation can be linked to support the management of specimens in the museum.

Given the rapid increase in the availability of high‐quality specimen and field images of plants, the capacity to utilize computer vision and image mining techniques to make automated taxonomic identifications, extract traits, and produce phenological scorings provides the field of convolutional neural networks and deep learning tremendous opportunities for applications in botany. Botella et al. (2018) review previous work with these tools, pointing out that recent progress with deep learning techniques has shown impressive recognition performance and that, when combined with mobile applications such as Pl@ntNet (https://identify.plantnet-project.org/), these techniques may contribute significantly to species distribution modeling (SDM), biodiversity monitoring, and the inclusion of citizen science observations within each of these domains. Their paper explores the use of automated identification in the absence of human validation for SDM, particularly the impact of the degree of uncertainty when training the MAXENT niche modeling approach. They evaluated five invasive species against a training set of 332,000 human‐validated plant images belonging to about 11,000 species. Their results suggest significant research challenges for using these types of data in SDMs, as well as for developing models for integrating citizen science observations into conservation management. Automated image mining is of continuing importance to botany and is a worthwhile avenue for further research.

Plant phenology (seasonal events such as leaf out, flowering, and fruiting) has complex effects on multiple levels of biological organization from individuals to ecosystems, and Yost et al. (2018) discuss the potential of herbarium specimens for addressing basic and applied research on plant phenology. Phenological shifts are key indicators of global change, and temporal mismatches in phenology may have important, even catastrophic effects on natural communities and agricultural systems. For example, such mismatches between plants and pollinators can quickly cause local extinctions, drive rapid evolutionary shifts, and cause billions of dollars of agricultural losses. Herbarium specimens are an excellent source of data for documenting changes in plant phenology (see review by Willis et al., 2017), but despite millions of specimens that could contribute to an understanding of historical phenology, inter‐year variation in phenology, and true shifts in phenology, the use of these data suffers from a lack of standardized scoring methods and definitions of phenological states. To date, phenological information has been captured in a herbarium specimen record in multiple ways, for example, in Darwin Core fields from ‘reproductiveCondition’ to ‘occurrenceRemarks,’ ‘organismRemarks,’ ‘dynamicProperties,’ or ‘fieldNotes.’ The lack of standardization in scoring and recording phenological data has limited large‐scale use of specimens for phenological study. Yost et al. propose a standardized methodology for scoring phenological characters from herbarium specimens that can be applied by researchers across herbaria, research groups, and means of data collection, including via citizen science, satellite imagery, and stationary cameras.

Herbaria for centuries have typically housed collections of not only plants, but also fungi. Despite current knowledge that fungi represent the sister group to animals and are not closely related to plants, many of the curatorial practices for fungi are similar to those for plants, and this similarity extends to digitization as well. Thiers and Halling (2018) describe the Macrofungi Collections Consortium (MaCC) and the development of MyCoPortal (http://mycoportal.org/portal/index.php) for serving digitized specimen information. MaCC digitized data from ~1.25 million specimens; including data contributed by the Microfungi Collections Consortium (http://www.microfungi.org/), the MyCoPortal database currently houses nearly 3.5 million specimens, as well as descriptions, illustrations, and observational records. The driving force behind development of MyCoPortal was production of a database to provide baseline data on the extent and distribution of macrofungal diversity, and the aggregated data have certainly accomplished this goal. Moreover, MyCoPortal has attracted the amateur mycological community from the United States, which comprises 80 clubs and 10,200 members. Together, professional and amateur mycologists, with the foundational data from MyCoPortal, are poised to produce a comprehensive mycoflora of North America, complete with DNA sequences, phenotypic descriptions, and images. Data from MyCoPortal have been used in taxonomic treatments, large‐scale phylogenetic analyses, ecological studies, and analyses of native versus invasive species and set the stage for a broad range of uses into the future.

Herbaria are reservoirs of both well‐documented specimens and undescribed diversity. New species are described each year from specimens that have been housed in collections for decades, if not centuries. However, the pace of such discovery is slow, especially for non‐angiosperms, and accelerating the process of discovery is expensive. von Konrat et al. (2018) explore the role of digitization in increasing accessibility to specimens, particularly in combination with citizen science efforts and online technology for uses beyond label transcription. The authors connect natural history collections to education and outreach through a citizen science tool based on the online Zooniverse platform. Their project, MicroPlants (http://microplants.fieldmuseum.org/), uses images of the liverwort genus *Frullania* and both a web‐based platform and an interactive touchscreen version to capture large data sets for taxonomic analysis, engage a diverse participant group in research, and expose the public to novel analytical approaches and the scientific process. MicroPlants has been used in informal science settings at the Field Museum and in formal educational venues in middle schools, high schools, and colleges and universities. The project has provided valuable data on both morphological variation in *Frullania* and the educational effectiveness of this citizen science platform. Noteworthy is the fact that preliminary analyses indicate that data provided by non‐experts were comparable to those generated by experts, supporting a role for citizen scientists in addressing authentic hypothesis‐driven research.

Data aggregators and publishers benefit significantly from knowing how their collections data are being used and attributed, the number of records and data sets being downloaded, the types of individuals who are finding these data useful, and the impact of projects for which the data are used. Usage metrics, in particular, help herbaria document value to institutional administrators as well as potential funders, and assist herbarium directors seek out and target underserved or expanding audiences. Cantrill (2018) summarizes tracked usage of nearly 900,000 records from the Royal Botanic Gardens Victoria, served through the Australasian Virtual Herbarium, and details trends in data usage since 2009. Queries were tracked in three broad categories, including general use, non‐research use, and scientific research use, with histories of how these categories and their subcategories have become more refined over the past decade. Cantrill points out that even with more highly resolved classifications, about one third of all queries still remain unclassified. He further notes that although the data give a glimpse of data use as reported by users, they do not provide a metric for understanding the impact of the projects for which they were downloaded. Future research must assess this issue if we are to understand and report the full impact of our collections.

This collection of papers provides a current snapshot of some of the issues surrounding the aggregation and use of digitized herbarium data and of some of the many possible uses of these data in research and education. However, the field is changing rapidly, with new tools for data mining, image analysis, and data tracking coming available at a rapid pace. The application of innovative analytic, algorithmic, and informatics approaches to centuries‐old specimens is revolutionizing the role of herbaria and other museum collections in modern biology.
